# A national study of clinical discussions about cannabis use among Veteran patients prescribed opioids

**DOI:** 10.1186/s42238-024-00221-3

**Published:** 2024-03-16

**Authors:** Tauheed Zaman, Dawn M. Bravata, Amy Byers, Erin Krebs, Samuel Leonard, Charles Austin, Friedhelm Sandbrink, Deborah S. Hasin, Salomeh Keyhani

**Affiliations:** 1 Addiction Recovery and Treatments Services, San Francisco VA Health Care System, 4150 Clement Street, #116F, San Francisco VA Medical Center, San Francisco, CA 94121 USA; 2 Department of Psychiatry and Behavioral Sciences, University of California, San Francisco, CA USA; 3 Richard L. Roudebush VA Medical Center, Indianapolis, IN USA; 4 Departments of Medicine and Neurology, Indiana University School of Medicine, Indianapolis, IN USA; 5 Regenstrief Institute, Indianapolis, IN USA; 6 Department of Medicine, University of California, San Francisco, CA USA; 7 Medical Service, San Francisco VA Health Care System, San Francisco, USA; 8 Center for Care Delivery and Outcomes Research, Minneapolis VA Health Care System, Minneapolis, MN USA; 9 Department of Medicine, University of Minnesota Medical School, Minneapolis, MN USA; 10 National Pain Management, Opioid Safety and Prescription Drug Monitoring Program, Veterans Health Administration, Washington, DC USA; 11 Department of Neurology, George Washington University, Washington, DC USA; 12 New York State Psychiatric Institute, New York, NY USA; 13 Department of Psychiatry, College of Physicians and Surgeons, Columbia University, New York, NY USA; 14 Division of General Internal Medicine, Medical Service, San Francisco Veterans Affairs Health Care System, San Francisco, CA USA; 15 Department of Medicine, University of California-San Francisco, San Francisco, CA USA

**Keywords:** Cannabis, Cannabis use in Veterans, Cannabis for pain, Cannabis documentation, Cannabis, And opioids

## Abstract

**Background:**

The Veterans Health Administration tracks urine drug tests (UDTs) among patients on long-term opioid therapy (LTOT) and recommends discussing the health effects of cannabis use.

**Objective:**

To determine the occurrence of cannabis-related discussions between providers and patients on LTOT during six months following UDT positive for cannabis, and examine factors associated with documenting cannabis use.

**Design:**

We identified patients prescribed LTOT with a UDT positive for cannabis in 2019. We developed a text-processing tool to extract discussions around cannabis use from their charts.

**Subjects:**

Twelve thousand seventy patients were included. Chart review was conducted on a random sample of 1,946 patients.

**Main measures:**

The presence of a cannabis term in the chart suggesting documented cannabis use or cannabis-related discussions. Content of those discussions was extracted in a subset of patients. Logistic regression was used to examine the association between patient factors, including state of residence legal status, with documentation of cannabis use.

**Key Results:**

Among the 12,070 patients, 65.8% (*N* = 7,948) had a cannabis term, whereas 34.1% (*N* = 4,122) of patients lacked a cannabis term, suggesting that no documentation of cannabis use or discussion between provider and patient took place. Among the subset of patients who had a discussion documented, 47% related to cannabis use for medical reasons, 35% related to a discussion of VA policy or legal issues, and 17% related to a discussion specific to medical risks or harm reduction strategies. In adjusted analyses, residents of states with legalized recreational cannabis were less likely to have any cannabis-related discussion compared to patients in non-legal states [OR 0.73, 95% CI 0.64–0.82].

**Conclusions:**

One-third of LTOT patients did not have documentation of cannabis use in the chart in the 6 months following a positive UDT for cannabis. Discussions related to the medical risks of cannabis use or harm reduction strategies were uncommon.

**Supplementary Information:**

The online version contains supplementary material available at 10.1186/s42238-024-00221-3.

## Introduction

Cannabis use is rising among Veterans, just as it is for the general population (Center for Behavioral Health Statistics and Quality, Substance Abuse and Mental Health Services Administration 2019a), (https://www.samhsa.gov/data/report/2019-nsduh-veterans). The legalization of cannabis has been associated with declining perceptions of risk towards use (Han et al. 2021) with divergent perceptions of risks across states with differing legal status. Residents of recreational legal states are more likely to believe that cannabis has medical benefits and less likely to think cannabis use has risks (Steigerwald et al. 2020). States with recreational legalization have seen an increased marketing of cannabis products (Ayers et al. 2019). Cannabis products are marketed for pain, insomnia, anxiety and a host of other indications (Lau et al. 2021; Azcarate et al. 2020a) Up to 60% of those using medical cannabis citing pain as the primary reason (Boehnke et al. 2022).

Although cannabis is marketed for pain, the relative risks and benefits are still unknown. A recent systematic review suggested that certain cannabinoid combinations may provide short-term improvement for neuropathic pain, but use was also associated with an increased risk of sedation and dizziness. (McDonagh et al. 2022) Additional studies suggest moderate benefit in chronic pain conditions more broadly (Aviram et al. 2021; McDonagh et al. 2022; Wang et al. 2021), and an association with opioid dose reduction among chronic pain patients in observational studies, though not in randomized controlled trials (Okusanya et al. 2020; Nielsen et al. 2022). The cannabis products studied are heterogeneous and the effects of long-term use are as yet unknown. Potential benefits must be considered alongside the risk of adverse effects of long-term cannabis use. Known risks of cannabis use include patients developing cannabis use disorder (Leung et al. 2020; Hasin et al. 2020; Hasin et al. 2016), with past year use disorder diagnosis increasing steadily among Veterans from 1.2% in 2016 to 4.4% in 2021 (Center for Behavioral Health Statistics and Quality, Substance Abuse and Mental Health Services Administration 2021; Hasin et al. 2022; Mannes et al. 2023).Cannabis use is also associated with poorer treatment outcomes for adults with depression (Bahorik et al. 2018) and reduced treatment engagement among patients with post-traumatic stress disorder (Bedard-Gilligan et al. 2018). Cannabis use is associated with psychosis (Marconi et al. 2016) and use of combustible cannabis may have cardiovascular risks given the known association between particulate matter and cardiovascular disease (Page et al. 2020). Notably, patients often report using cannabis products for symptoms such as insomnia, anxiety, and depression (Azcarate et al. 2020), though for psychiatric conditions is not currently supported by the American Psychiatric Association (American Psychiatric Association 2019).

In 2014 the Veterans Health Administration (VHA) recommended that pain patients prescribed opioids receive annual urine drug testing (UDT) (Veterans Health Administration Opioids Safety Initiative 2021; U.S. Department of Veterans Affairs Pain Management and Opioid Safety Educational Guide 2014) and began tracking this metric, and in 2017 recommended that providers discuss medical cannabis use with their patients and document its use (Directive 1315) (Office, V. W. S. n.d.). Thus, national data from the Veterans Affairs (VA) Health Care System, the largest integrated US healthcare system with a large proportion of patients seeking treatment for pain, provides a unique opportunity to examine whether patients with chronic pain who test positive for cannabis receive subsequent clinical discussion during outpatient visits given the many potential impacts of cannabis use on health (National Academies Press 2017).

This national, population-based study used UDT data to identify patients on long-term opioid therapy (LTOT) who also used cannabis and examined whether providers documented a discussion of cannabis use in the electronic medical record during the 6 months following UDT. The study also examines factors associated with documentation of cannabis use, including patient demographics, state-level legalization status, substance use and other psychiatric co-morbidities.

## Methods

### Data and participants

**Fig. 1:**
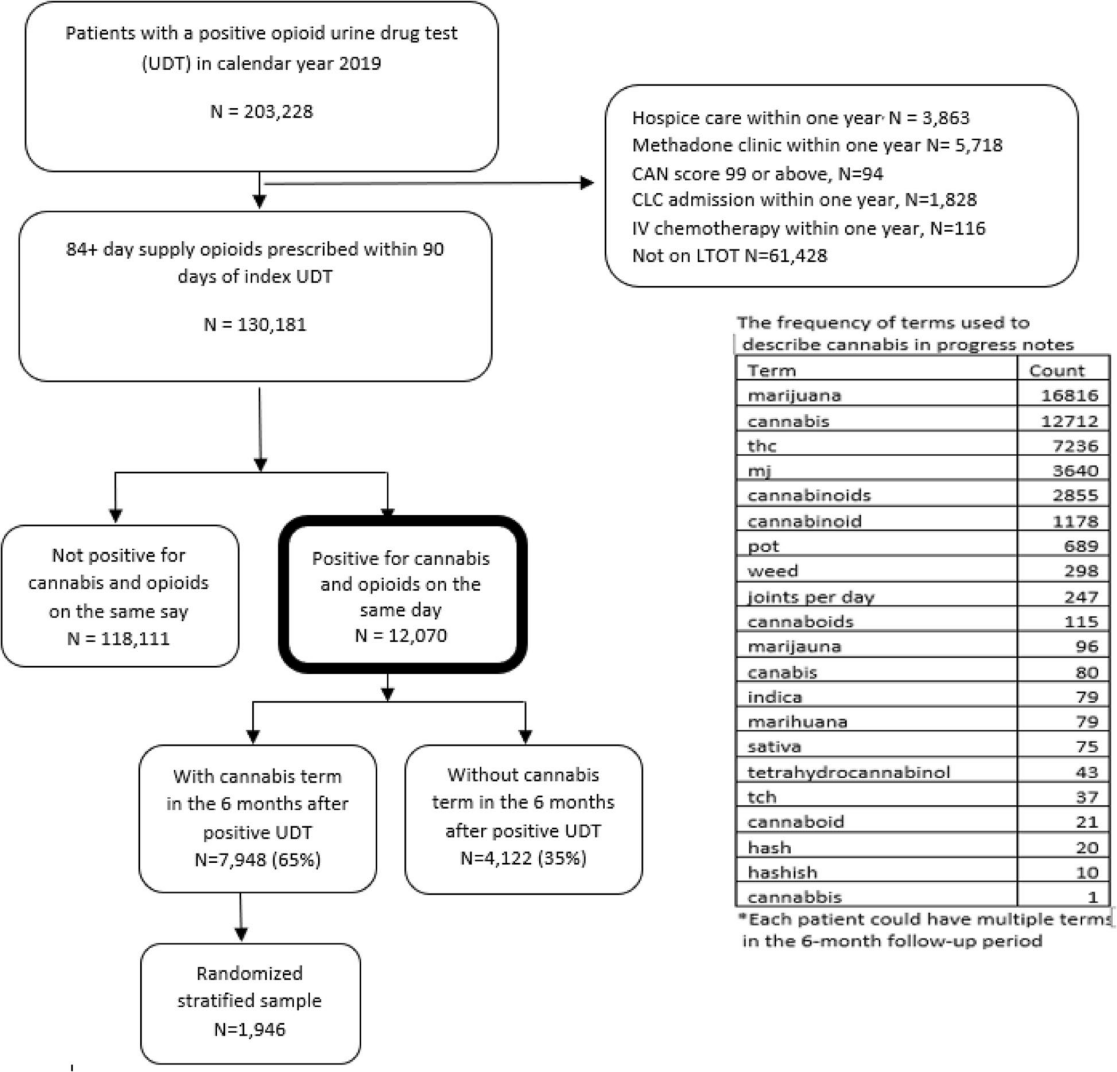
Proportion of Veterans on long-term opioids and cannabis in their urine drug test (UDT) that has a mention of cannabis use in their provider notes within the 6-month period after the index UDT

Using data from the VHA Corporate Data Warehouse (CDW) (Veterns Health Administration Corporate Data Warehouse n.d.), we identified all Veterans aged 18 or older who received a UDT in primary care in all 50 states in 2019, which was the last year during which such data was gathered as part of a separate study (Keyhani et al. 2022a) We then limited the sample to the first UDT in primary care received by each patient in 2019. As our goal was to ensure patients in the cohort were not using cannabis for end of life or palliative conditions and were community dwelling, we excluded patients who had received hospice care, were in a nursing home, or VA Community Living Center (CLC), had a very high Care Assessment Need score (CAN score) signifying limited life expectancy, and those who were receiving inpatient chemotherapy. We further limited the cohort to patients who had received 84 + days’ supply of opioids prescribed in the prior 90 days (adults on long-term opioid therapy [LTOT]) and additionally were positive with a UDT for cannabis use on the same day, which resulted in a final sample of 12,070 patients (Fig. 1 describes the study flowchart). We focused on patients on LTOT as these patients are at high risk for adverse outcomes.

### Approvals

The institutional review board (IRB) of the University of California, San Francisco approved this study.

### Text processing to identify mentions of cannabis use in the electronic medical record

To capture a mention of cannabis use, we created a dichotomous variable (yes/no) defining presence of cannabis terms in charts for study participants in the electronic medical record during the 6 months after index UDT. This was achieved as follows: a list of commonly used terms that describe cannabis (e.g., marijuana, cannabis, hashish) were generated by the investigative team. The list of terms was expanded by reviewing charts of patients with a positive UDT and looking for new ways to describe cannabis. In addition, three abstractors using the national VA electronic health record system independently reviewed a sample of 100 charts of patients who had a UDT positive for cannabis but did not have a cannabis term. These abstractors then identified further terms that could be used to describe cannabis use, including common misspellings of terms that were noted by the abstractors in the medical record. (see Supplement for more details on term development and modifications made to text search to eliminate false positives). We repeated this step until we found no new terms. Using the VA Informatics and Computing Infrastructure (VINCI), we searched for these original terms in all the outpatient notes for each patient in the 6-month period after the index UDT (including the day of the drug test). The final list of terms and their frequency is available in Fig. 1.

### Extracting provider discussion and actions around cannabis use among patients with cannabis term in chart

To examine provider discussions around cannabis use, we selected a random sub-sample of patients with a cannabis term from each facility (*N* = 1,946, Fig. 1). We ensured that at least 25 patients from each facility were included to improve the generalizability of the sub-sample. If a facility did not have 25 patients in the sub-sample, all cases from that facility were included. This also ensured we had adequate representation from states with approved legal recreational cannabis (RL), medically legal cannabis (ML), and non-legal cannabis (NL), as providers who reside in states with different legal status may have differing approaches to discussions around cannabis use. Using the text processing algorithm described above, we then searched notes from visits with primary care, geriatric, mental health, and substance use clinics in the 6-month period after a positive UDT. We searched notes from these clinical encounters, as a discussion around cannabis use after a positive urine drug screen was more likely in these settings compared to visits to other services (e.g., surgery, podiatry, and other medical subspecialty services).

We extracted the cannabis terms and included 90 characters before and after each term. We extracted 90 characters because we found with fewer characters, we could not reliably characterize the discussions or provider actions. All text snippets pertaining to cannabis were extracted for each patient for the 6-month period after the index UDT to ensure adequate time was available for providers to review the results, counsel patients and/or take actions related to the results. Two abstractors independently reviewed each cannabis-related text snippet. Ten percent of patients were reviewed in duplicate. Because presence of a cannabis term could refer to use and not necessarily a discussion, each snippet of text was categorized as pertaining to a discussion/provider action surrounding cannabis use or not. We further categorized the snippets that pertained to a discussion as follows: a documented discussion, or no discussion but a documented provider action. Documentation of discussions were further characterized: (1) discussions around patient reported cannabis use for a medical reason, (2) discussion of medical risks and/or harm reduction strategies (e.g., not smoking cannabis), (3) other types of discussions focused on legality of use or VA policy. We defined “action” as changing dose or stopping opioids, increased monitoring of use patterns with a UDT, specialist referral for treatment of substance use, or a letter to the patient about the presence of cannabis in the UDT. If neither abstractor could characterize the snippet based on available extracted text, they were instructed to review the full medical record. If the snippet could not be characterized by full chart examination it was reviewed by the investigative team (TZ, SK, AB, DMB) and was adjudicated by consensus. Disagreements between the abstractors were also adjudicated by the investigative team. Overall agreement among the 10% of cases reviewed in duplicate was 95.7%.

### The association of patient characteristics with documentation of cannabis use

The presence of a cannabis term in the chart signified that a provider documented cannabis use or had a discussion around cannabis use with the patient. We examined the association of patient characteristics with documentation of cannabis use in the chart.

#### Dependent variable

The main dependent variable was presence of a cannabis term in the progress notes in the 6-month period after a UDT positive for cannabis.

#### Independent variables

The legal status of each Veteran’s state of residence at the time of the UDT in 2019 was used to classify the legal status of the state of residence of each Veteran.

All other measures were extracted from the VA CDW within the 2-years prior to the index UDT (VA Informatics and Computing Infrastructure n.d.) and included demographic factors (age, race, gender, ethnicity and marital status), health behaviors (tobacco use, unhealthy alcohol use and other substance use disorders), mental health conditions and measures of socioeconomic status (e.g., housing). We used elevated AUDIT-C scores (Higgins-Biddle and Babor 2018) to identify adults’ hazardous alcohol use (score ≥ 4 for women and ≥ 5for men as recommended by the VHA) (Bush et al. 1998; Veterans Administration n.d.). We used a previously validated algorithm to identify current tobacco use. (Barnett et al. 2014) The algorithm is based on multiple sources of data including data from an electronic clinical reminder that queries patients on tobacco use in primary care and use of tobacco cessation services and counseling.

We used International Classification of Disease-10 (ICD-10) codes to identify patients with alcohol use disorder, drug use disorder, anxiety, depression, post-traumatic stress disorder, bipolar disorder, psychosis, schizophrenia, and schizoaffective disorder. Veterans were classified as “marginally housed” if an ICD-10 code related to housing insecurity or homelessness was present or if they received housing services.

We described the sample using bivariate analyses of baseline characteristics by presence of a cannabis term in the notes. Characteristics were summarized using frequencies and proportions and compared as a function of cannabis use using chi-square tests for categorical variables and t-test for continuous variables. Findings were considered statistically significant at a *P* < 0.05. We examined the associations between demographic, behavioral, and clinical factors, and presence of any cannabis related documentation in the electronic health record using multivariable logistic regression modeling. Estimates were statistically significant if the confidence intervals of the odds ratios (ORs) did not include the null value. We used RStudio version 1.4.17 for all analyses.

## Results

### Documentation of cannabis use and discussions around cannabis use

The final list of terms used to identify cannabis use in the charts included 21 terms (Fig. 1). Of these 21 terms, two (marijuana and cannabis) were the most used.

Among the 12,070 Veterans, 65% (*N* = 7,948) had documentation of a cannabis term in the notes in the 6 months after the UDT and 35% (*N* = 4,122) did not have any cannabis-related term. Among the 4,122 patients who had no documentation of cannabis use, approximately 95% (*N* = 3,905) had a visit with a primary care provider, mental health provider or substance use provider. Among the 3905 patients with such a visit, 77% (*N* = 3,022) were seen by a primary care provider, 22% (*N* = 844) seen by both primary care and by a mental health or substance use provider, and 0.99% (*N* = 39) seen only by a mental health or substance use provider.

Among the 1,946 random stratified patients identified using text processing as having a cannabis term in their charts, 1,935 (> 99%) were verified as having an actual cannabis term. Among these patients, 1,557 (80.5%) had terms referring to a provider having a discussion or taking an action in response to the cannabis positive UDT. In the remaining 378 (19.5%) of cases, the mention of cannabis was a documentation of use without a clear discussion or action completed thereafter (Table 1).

**Table 1:** Provider discussions and actions related to cannabis detected via text processing, stratified by state-level legal status

	**All states** **(N, %)**	**RL**^a^** states** **(N, %)**	**ML**^a^** states** **(N, %)**	**NL**^a^** states** **(N, %)**	***p***
**Total patients**	**1,935**	**517**	**848**	**570**	
Documentation of cannabis use only	378 (19.5%)	132 (25.5%)	153 (18.0%)	93 (16.3%)	< 0.01
Discussion or action documented in response to patient reported cannabis use	1,557 (80.5%)	385 (74.5%)	695 (82.0%)	477 (83.7%)	< 0.01
**Patients with a discussion or action documented in response to cannabis use**	**1557**	**385**	**695**	**477**	
Documentation of provider action^b^	202 (13.0%)	43 (11.2%)	73 (10.5%)	86 (18.0%)	< 0.01
Documentation of discussion without provider action^b^	1,355 (87.0%)	342 (88.8%)	622 (89.5%)	391 (82.0%)	< 0.01

^a^*RL* Legal recreational marijuana, *ML* Legal medical marijuana, *NL* non-legal marijuana

^b^Provider actions included changing opioid dosage or prescription, stopping opioid prescription, increased monitoring, specialist referral, letter about UDS results, provider endorsement/recommendation, other

**Fig. 2:**
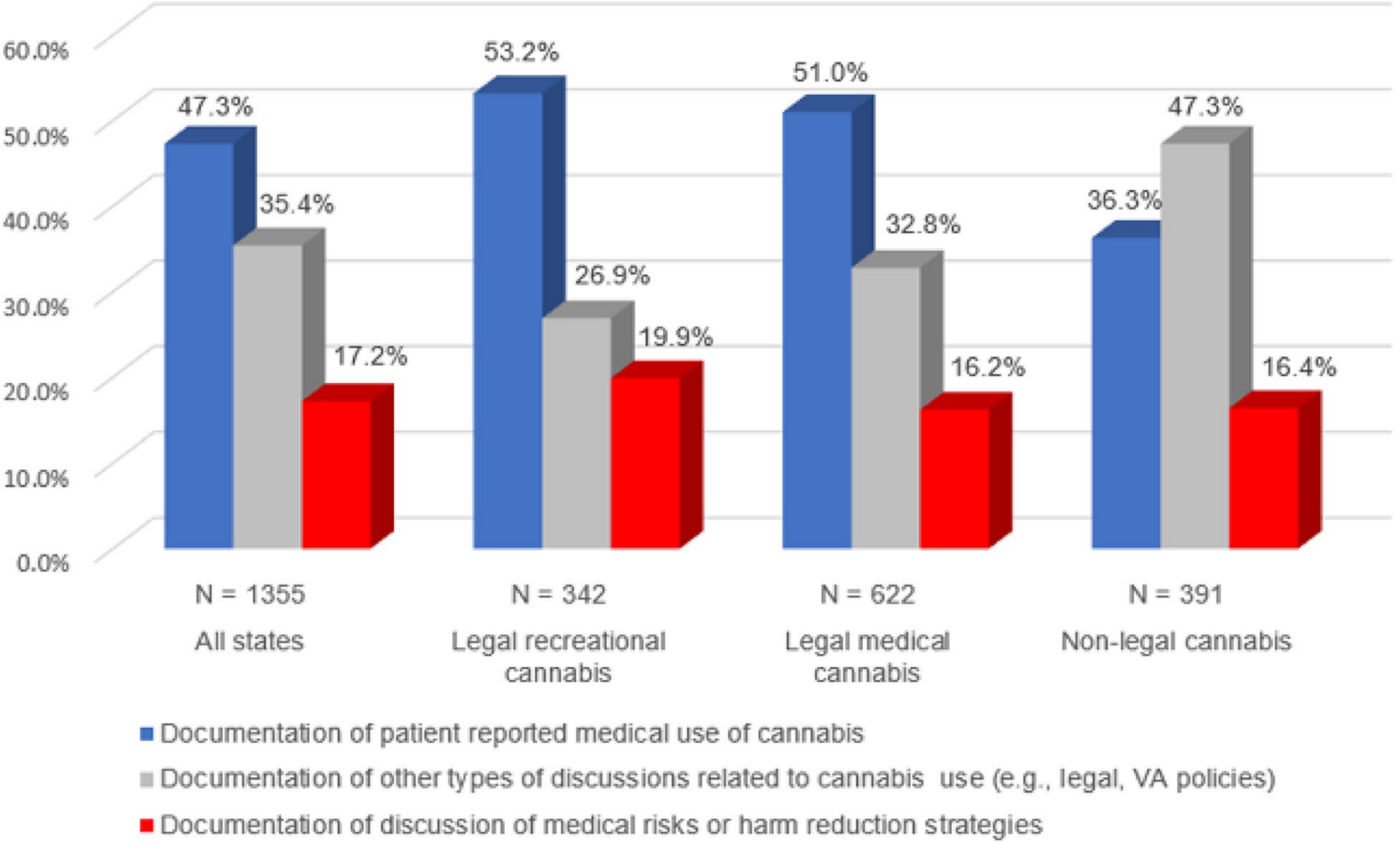
Content of cannabis-related discussions in patient charts stratified by state-level legalization status

Among the cases where a discussion or action was documented, 1,355 (87%) were a discussion and 202 (13%) were a provider action. Among those who had a discussion documented, 641 (47%) were a discussion of cannabis use for medical reasons where patients disclosed medical use to their providers, 481 (35%) were a discussion of VA policy or legal issues and 233 (17%) were a discussion specific to medical risks or harm reduction strategies. Patients in RL states were less likely than those in ML or NL states to have both documentation and action related to cannabis (*p* < 0.01). Patients in RL states were more likely to have a discussion of medical risks or harm reduction strategies, while those in ML and NL states were more likely to discuss non-medical issues (e.g., legal and VA policy; Fig. 2).

#### Baseline characteristics of the overall cohort

Mean age differences among those with and without documentation of cannabis use was small (61.7 (10.5) vs 62.9 (9.9) years, *p* < 0.01; Table 2). Racial and ethnic differences among those with and without cannabis documentation was also small; compared to adults without documentation, those with cannabis terms in the notes were more likely to be Hispanic (5.9% vs 4.5%, *p* < 0.01). Cannabis documentation was more common among those who were homeless/receiving housing services (8.4% vs 6.6%, *p* < 0.01). Adults with cannabis terms documented in the notes were more likely to have a diagnosis of alcohol use disorder (10.9% vs 7.6%, *p* < 0.01), have an elevated AUDIT C score (5.4% vs 4.3%, *p* = 0.012), non-alcohol drug use disorder (15.6% vs 8.5%, *p* < 0.01), anxiety (20.2% vs 14.1%, *p* < 0.01), depression (37.5% vs 26.1%, *p* < 0.01), PTSD (30.3% vs 21.3%, *p* < 0.01), bipolar disorder (5.6% vs 2.4%, *p* < 0.01), and psychotic disorders (2.5% vs 1.1%, *p* < 0.01) compared to those without documented cannabis terms.

**Table 2:** Characteristics of Veterans 18 and older on LTOT with positive urine drug screen for cannabis in 2019 with and without documentation related to cannabis use

Patient Characteristic	Overall (N, %)	No documentation of cannabis use in outpatient notes (N, %)	Documentation of cannabis in outpatient notes (N, %)	*p*
	12,070	4,122	7,948	
Mean age in years (SD)^a^	62.1 (10.3)	62.9 (9.9)	61.7 (10.5)	< 0.01
Age (years)				< 0.01
18–44	983 (8.1)	278 (6.7)	705 (8.9)	
45–64	5,355 (44.4)	1,786 (43.3)	3,569 (44.9)	
65–74	5,1000 (42.3)	1,797 (43.6)	3,303 (41.6)	
75–84	570 (4.7)	237 (5.7)	333 (4.2)	
85 or older	62 (0.5)	24 (0.6)	38 (0.5)	
Male Gender	11,280 (93.5)	3,890 (94.4)	7,390 (93.0)	0.04
Race				0.028
American Indian or Alaska Native	166 (1.4)	41 (1.0)	125 (1.6)	
Asian	47 (0.4)	13 (0.3)	34 (0.4)	
Black or African American	1,518 (12.6)	542 (13.1)	976 (12.3)	
More than one race	139 (1.2)	59 (1.4)	80 (1.0)	
Native Hawaiian or other Pacific Islander	105 (0.9)	35 (0.8)	70 (0.9)	
Unknown	805 (6.7)	284 (6.9)	521 (6.6)	
White	9,290 (77.0)	3,148 (76.4)	6,142 (77.3)	
Hispanic or Latino	655 (5.4)	185 (4.5)	470 (5.9)	< 0.01
Married	5,442 (45.1)	1,850 (44.9)	3,5920 (45.2)	0.75
Currently Tobacco smoker	1,363 (11.3)	452 (11.0)	911 (11.5)	0.43
Alcohol use disorder	1,182 (9.8)	312 (7.6)	870 (10.9)	< 0.01
Elevated Audit C	610 (5.1)	179 (4.3)	431 (5.4)	0.012
Drug use disorder	1,594 (13.2)	351 (8.5)	1,243 (15.6)	< 0.01
Anxiety	2,186 (18.1)	582 (14.1)	1,604 (20.2)	< 0.01
Depression	4,054 (33.6)	1,074 (26.1)	2,980 (37.5)	< 0.01
PTSD	3,289 (27.2)	878 (21.3)	2,411 (30.3)	< 0.01
Bipolar disorder	547 (4.5)	100 (2.4)	447 (5.6)	< 0.01
Psychosis/Schizophrenia, Schizoaffective Disorder	248 (2.1)	47 (1.1)	201 (2.5)	< 0.01
Homeless/ Receipt of Housing Services	940 (7.8)	270 (6.6)	670 (8.4)	< 0.01
Legal status of state of residence				< 0.01
Recreationally legal	6,180 (51.2)	2,385 (57.9)	3,795 (47.7)	
Medical legal	4,284 (35.5)	1,236 (30.0)	3,048 (38.3)	
Non-legal	1,606 (13.3)	501 (12.2)	1,105 (13.9)	

^a^Standard deviation

#### Multivariable regression results examining association of patient characteristics and documentation of cannabis use in the chart

Younger patients 18 to 44 years in age [AOR 1.34, 95% CI 1.07–1.66] and those 45–64 years in age [AOR 1.18, 95% CI 1.00–1.41] were more likely to have cannabis use documented in the chart compared to older adults (Table 3). Hispanic adults were also more likely to have cannabis use documented [AOR 1.21, 95% CI 1.01–1.45]. Adults with a diagnosis of alcohol use disorder [AOR 1.17, 95% CI 1.01–1.36], non-alcohol drug use disorder [AOR 1.59, 95% CI (1.39–1.81)], anxiety [AOR 1.15, 95% CI 1.02–1.28], depression [AOR 1.42, 95% CI 1.29–1.55], PTSD [AOR 1.32, 95% CI 1.20- 1.45], bipolar disorder [AOR 1.90, 95% CI 1.52–2.39], and psychotic disorders [AOR 1.76, 95% CI 1.26–2.44)] were more likely to have cannabis use documented in the chart. Residents of states with legalized recreational cannabis were less likely to have documentation of cannabis use in the chart than patients in non-legal states [AOR 0.73 95% CI 0.64–0.82]. There was no difference in documentation of cannabis use between medically legal and non-legal states [AOR 1.10 95% CI (0.97, 1.25)].

**Table 3:** Factors associated with the documentation of cannabis use among Veterans 18 and older on LTOT in 2019

	**Unadjusted OR (95% CI)** **for presence of a note**	**Adjusted OR (95% CI) for presence of a note**	***p***
Age (years)			
18–44	1.78 (1.44, 2.21)	1.34 (1.07, 1.66)	0.01
45–64	1.41 (1.18, 1.67)	1.18 (1.00, 1.41)	0.06
65–74	1.29 (1.09, 1.54)	1.15 (0.97, 1.37)	0.11
> 75		reference	
Male Gender	0.79 (0.67, 0.93)	0.96 (0.81, 1.13)	0.61
Race			
Black		reference	
White	1.08 (0.97, 1.22)	1.11 (0.99, 1.25)	0.09
Other	1.07 (0.91, 1.25)	1.09 (0.93, 1.29)	0.29
Hispanic or Latino	1.34 (1.12, 1.60)	1.21 (1.01, 1.45)	0.04
Married	1.01 (0.94, 1.09)	0.99 (0.92, 1.07)	0.83
Current Tobacco smoker	1.05 (0.93, 1.19)	0.97 (0.86, 1.10)	0.66
Alcohol use disorder	1.50 (1.31, 1.72)	1.17 (1.01, 1.36)	0.03
Elevated Audit C	1.26 (1.05, 1.52)	1.18 (0.97, 1.43)	0.09
Drug use disorder	1.99 (1.76, 2.26)	1.59 (1.39, 1.81)	< 0.01
Anxiety	1.54 (1.39, 1.71)	1.15 (1.02, 1.28)	0.02
Depression	1.70 (1.57, 1.85)	1.42 (1.29, 1.55)	< 0.01
PTSD	1.61 (1.47, 1.76)	1.32 (1.20, 1.45)	< 0.01
Bipolar disorder	2.40 (1.92, 3.02)	1.90 (1.52, 2.39)	< 0.01
Psychosis/Schizophrenia, Schizoaffective Disorder	2.25 (1.63, 3.17)	1.76 (1.26, 2.44)	< 0.01
Homeless/receipt of housing services	1.31 (1.13, 1.53)	1.16 (0.99, 1.36)	0.06
Legalization status of state of residence			
Recreationally legal state	0.72 (0.64, 0.81)	0.73 (0.64, 0.82)	< 0.01
Medically legal state	1.12 (0.98, 1.27)	1.10 (0.97, 1.25)	0.15
Non-legal		reference	

## Discussion

One-third of patients had no documentation of cannabis use in their charts in the 6-month period following a positive UDT, despite the majority being seen by primary care providers, mental health/substance use providers, or both. This finding was notable given the increasing prevalence of cannabis use among US adults (Center for Behavioral Health Statistics and Quality, Substance Abuse and Mental Health Services Administration 2019b) and cannabis use disorder among Veterans with pain. (Mannes et al. 2023) However, there are currently no standard screening tools or treatment guidelines similar to those the VA recommends for substances like alcohol (VA Direction 1120.05 n.d.) and tobacco (VA Directive 1056 n.d.), which may hinder providers from having meaningful conversations about cannabis with their pain patients who are using both cannabis and opioids.

Fewer Veterans in RL states received discussions of cannabis compared to those in ML or NL states (Table 1). This may occur because many healthcare providers are uncomfortable or inconsistent in discussing cannabis use with their patients (Brooks et al. 2017), and these inconsistencies may be exacerbated by differing legal status between states. Legalization and increased availability of cannabis are both associated with a reduced perception of risk (Mennis et al. 2023; Fataar et al. 2021; Levy et al. 2021). Therefore, in RL states both patients and providers may perceive cannabis use as being less risky because of this “normalizing” effect, leading to fewer clinical conversations and actions taken overall. Physicians in RL states may be less likely to consider cannabis use in the same category as nonlegal substances (e.g., illicit opioids and stimulants), and share patients’ opinions regarding pain benefit, leading to reduced overall discussion of cannabis. The differences between states raises the possibility that patients who are particularly vulnerable to cannabis-related harms, such as those with histories of addiction (Leung et al. 2020) and of psychotic disorders (Hasan et al. 2020), may be less likely to receive discussion of cannabis in RL states.

Of note, while patients in RL states were less likely to have documentation of any discussion of cannabis, if a discussion did take place, it was more likely to include medical risks of cannabis or harm reduction strategies (Fig. 2). This may occur because a subset of providers in RL states may have more experience working with patients using cannabis and discussing related medical risks or harm reduction compared to ML and NL states. In contrast, providers in ML and NL states may have more experience with restrictive cannabis laws and discussion of non-medical policy or legal issues with patients. Notably, the VHA does not allow providers to authorize medical cannabis use, and therefore all patients with cannabis use received it from non-VA sources. Nor does the VA provide training specific to cannabis-related discussion and documentation to its clinicians. More guidance on a standardized approach to discussing cannabis with these patients and documenting such clinical conversations may reduce the differences across facilities, particularly in a nationalized service such as the VHA.

Veterans with concurrent substance use and other mental health issues were overall more likely to receive discussions of cannabis. This appears clinically appropriate given the association between cannabis use and other substance use (Blanco et al. 2016). Cannabis use among adults is associated with increased risk of tobacco, alcohol, and drug use. (Jeffers et al. 2021; Keyhani et al. 2022b; Agrawal et al. 2012; Peters et al. 2012) Patients receiving treatment for substance use disorders may be more likely to discuss cannabis use with their providers or receive clinical screening, leading to higher rates of discussion of cannabis and documentation. Patients with a history of psychotic disorders may be more likely to receive discussion of cannabis given provider knowledge of the risk of psychotic outcomes associated with cannabis use (Hasan et al. 2020). In addition, there is insufficient evidence to suggest that plant-based cannabis use or cannabinoids has benefits among patients with mental health conditions (Whiting et al. 2015; Stanciu et al. 2021; Hill et al. 2022; Black et al. 2019) and potential negative effects on treatment engagement (Bedard-Gilligan et al. 2018). Some patients report attempting managing mental health symptoms with cannabis products (Kalaba and Ware 2022), and particularly cannabidiol (CBD) (Wieckiewicz et al. 2022). Therefore, providers must assist patients with the management of relevant underlying symptoms while providing education about the potential effects of cannabis on mental health.

This study has limitations that warrant comment. The population is predominantly male and older in age, and findings may not generalize to other populations. Although a substantial number of Veterans with combined cannabis and opioid use had no mention of cannabis use documented in the chart in the 6-month period after a positive UDT, absence of documentation does not necessarily mean a lack of discussion. It is possible providers did engage in discussion of cannabis and discussions and/or actions that were not documented. Our studies report data from 2019, and there may have been changes in documentation rates related to cannabis since this time period. Future studies may examine the discussion of other substances such as alcohol and tobacco use in comparison to cannabis, and determine whether there are similar trends in the documentation of substance use more broadly among Veterans in the primary care setting.

## Conclusion

One-third of opioid-prescribed patients in VHA who used cannabis did not have documentation of cannabis use in the chart in the 6 months following a positive UDT for cannabis. Patients prescribed opioids who reside in RL states were less likely overall to receive cannabis-related discussions. Among the small subset that had a discussion with their provider about cannabis use in RL, a discussion of medical risks and or harm reduction strategies was more common compared to those in ML and NL states. Those with histories of substance use and other mental health issues were more likely to have cannabis documented in the chart. Standardized screening tools or treatment guidelines will be important to implement in practice to support providers having meaningful discussions about cannabis use with their patients.

## Supplementary Information

**Supplementary Material 1.**

## Data Availability

The data can be accessed by obtaining access to the VA Informatics Computing Infrastructure according to the Veterans Health Administration policies and procedures.
