# Supplementary material for: A national study of clinical discussions about cannabis use among Veteran patients prescribed opioids
Source: J Cannabis Res. 2024 Mar 16;6:12. doi: 10.1186/s42238-024-00221-3 (PMC10943860; doi:10.1186/s42238-024-00221-3)
Supplement: Supplementary file 1 — Supplementary Material 1. [file 42238_2024_221_MOESM1_ESM.docx]

**A national study of clinical discussions about cannabis use**

**among Veteran patients prescribed opioids**

**Supplementary Materials**

**AUTHORS**: Tauheed Zaman^1,2^, Dawn M. Bravata^3,4,5^, Amy Byers^2,6,7^, Erin Krebs^8,9^, Samuel Leonard^7^, Charles Austin^3^, Friedhelm Sandbrink^10,11^, Deborah S Hasin^12,13^, Salomeh Keyhani^7,14,15^

**AUTHOR AFFILIATIONS**:

1. Addiction Recovery and Treatments Services, San Francisco VA Health Care System, San Francisco, CA
2. Department of Psychiatry and Behavioral Sciences, University of California, San Francisco, CA
3. Richard L. Roudebush VA Medical Center, Indianapolis, IN
4. Departments of Medicine and Neurology, Indiana University School of Medicine, Indianapolis, IN
5. Regenstrief Institute, Indianapolis, IN
6. Department of Medicine, University of California, San Francisco, CA
7. Medical Service, San Francisco VA Health Care System
8. Center for Care Delivery and Outcomes Research, Minneapolis VA Health Care System, Minneapolis, MN
9. Department of Medicine, University of Minnesota Medical School, Minneapolis, MN
10. National Pain Management, Opioid Safety and Prescription Drug Monitoring Program, Veterans Health Administration, Washington DC
11. Department of Neurology, George Washington University, Washington DC
12. New York State Psychiatric Institute, New York, NY
13. Department of Psychiatry, College of Physicians and Surgeons, Columbia University, New York, NY
14. Division of General Internal Medicine, Medical Service, San Francisco Veterans Affairs Health Care System, San Francisco, CA
15. Department of Medicine, University of California-San Francisco, CA

**Identifying terms that can be used to describe cannabis use:**

A list of possible terms that can be used to describe cannabis (e.g., marijuana, cannabis, hashish) were generated by the investigative team. We searched the notes of patients who had a positive urine drug test an iteratively expanded the list of terms used to describe cannabis. The preliminary terms identified included (marijuana, cannabis, thc, mj, cannabinoids, pot, weed and joints per day)

We then identified a random sample of 100 cases from the cohort with positive urine drug test for cannabis, that had no cannabis term listed above from index date to 6 months after index. Abstractors conducted chart review in the 6-month period after negative urine drug test to find any mention of cannabis use in notes to see if there are any mentions of cannabis that were missed in the first step. We then repeated this step until we identified no new terms.

Positive cannabis use terms: (a space was inserted before and a space after each term in the search)

marijuana

cannabis

thc

cannabinoids

cannabinoid

pot

remove “neti pot”

weed

remove “weed eater”

remove “horny goat weed”

joints per day

cannaboids

maraijuana

canabis

indica

marihuana

sativa

tetrahydrocannabinol

tch

- - - 1. remove “tch XXXX XXX”

cannaboid

hash

hashish

cannabbis

The search included a space before and a space after cannabis. Through iterative chart review we found that the term pot, weed and the incorrect spelling for THC (TCH) had false positives. We removed cases in which the text search found the word pot next to neti and weed next to eater. Finally, TCH followed by a series of number (referring to a lab code) reduced the false positives.

The table below demonstrates the frequency of the terms after modifications to the search above were implemented. We searched for these terms in the medical record and extracted the text with 90 characters before and 90 characters afterwards into database for review by the chart review team.

**Table S1. The frequency of terms in the notes in the sample***

| **Term** | **Count of Terms in Medical Record** |
| --- | --- |
| marijuana | 16816 |
| cannabis | 12712 |
| thc | 7236 |
| mj | 3640 |
| cannabinoids | 2855 |
| cannabinoid | 1178 |
| pot | 689 |
| weed | 298 |
| joints per day | 247 |
| cannaboids | 115 |
| marijauna | 96 |
| canabis | 80 |
| indica | 79 |
| marihuana | 79 |
| sativa | 75 |
| tetrahydrocannabinol | 43 |
| tch | 37 |
| cannaboid | 21 |
| hash | 20 |
| hashish | 10 |
| cannabbis | 1 |

*Each chart could have multiple terms and multiple different terms,

the frequency does not represent unique patients
